# Efficacy of positive end-expiratory pressure titration after the alveolar recruitment manoeuvre in patients with acute respiratory distress syndrome

**DOI:** 10.1186/cc7725

**Published:** 2009-02-24

**Authors:** Jin Won Huh, Hoon Jung, Hye Sook Choi, Sang-Bum Hong, Chae-Man Lim, Younsuck Koh

**Affiliations:** 1 Department of Pulmonary and Critical Care Medicine, Inje University Ilsan Paik Hospital, Daehwa-dong, Goyang-si, 411-706, Korea; 2 Department of Pulmonary and Critical Care Medicine, Dongguk University, College of Medicine, Seokjang-dong, Gyeongju-si, 780-714, Korea; 3 Department of Pulmonary and Critical Care Medicine, Asan Medical Center, University of Ulsan College of Medicine, 388-1 Pungnap-dong, Seoul 138-736, Korea

## Abstract

**Introduction:**

In acute respiratory distress syndrome (ARDS), adequate positive end-expiratory pressure (PEEP) may decrease ventilator-induced lung injury by minimising overinflation and cyclic recruitment-derecruitment of the lung. We evaluated whether setting the PEEP using decremental PEEP titration after an alveolar recruitment manoeuvre (ARM) affects the clinical outcome in patients with ARDS.

**Methods:**

Fifty-seven patients with early ARDS were randomly assigned to a group given decremental PEEP titration following ARM or a table-based PEEP (control) group. PEEP and inspired fraction of oxygen (FiO_2_) in the control group were set according to the table-based combinations of FiO_2 _and PEEP of the ARDS network, by which we aimed to achieve a PEEP level compatible with an oxygenation target. In the decremental PEEP titration group, the oxygen saturation and static compliance were monitored as the patients performed the ARM along with the extended sigh method, which is designed to gradually apply and withdraw a high distending pressure over a prolonged period, and the decremental titration of PEEP.

**Results:**

The baseline characteristics did not differ significantly between the control and decremental PEEP titration groups. Initial oxygenation improved more in the decremental PEEP titration group than in the control group. However, dynamic compliance, tidal volume and PEEP were similar in the two groups during the first week. The duration of use of paralysing or sedative agents, mechanical ventilation, stay in the intensive care unit and mortality at 28 days did not differ significantly between the decremental PEEP titration and control groups.

**Conclusions:**

The daily decremental PEEP titration after ARM showed only initial oxygenation improvement compared with the table-based PEEP method. Respiratory mechanics and patient outcomes did not differ between the decremental PEEP titration and control groups.

**Trial registration:**

ClinicalTrials.gov identifier: ISRCTN79027921.

## Introduction

Two recent randomised controlled trials involving patients with acute lung injury (ALI) or acute respiratory distress syndrome (ARDS) demonstrated that mortality can be reduced significantly by setting a low tidal volume (V_T_) [1] and by setting both a low V_T _and adequate positive end-expiratory pressure (PEEP) levels titrated by pressure-volume curves [2,3]. However, this strategy favours further lung collapse or derecruitment, especially when used with a high inspired fraction of oxygen (FiO_2_) [4]. The importance of opening the lung and keeping it open seems increasingly significant in this era of lung-protective ventilatory support because the use of a small V_T _for this strategy may worsen progressive lung collapse. Moreover, the alveolar and systemic inflammatory responses can be attenuated by minimising overinflation and cyclic recruitment–derecruitment of the lung by reducing V_T _and increasing PEEP in ARDS [5].

The best method of setting optimal PEEP after recruitment to prevent recollapse is still a matter of debate [6]. In the ALVEOLI study, mortality rates and the number of ventilator-free days did not differ significantly between the lower- and higher-PEEP study groups [7]. This result may have reflected the use of the same PEEP level (combination of FiO_2 _and PEEP) for the heterogeneous patient group and an inappropriately high PEEP in the nonrecruiters, which may have resulted in overdistension. Gattinoni and colleagues reported that the percentage of potentially recruitable lung tissue may be different in patients with ARDS and that the use of a higher PEEP level in patients with a lower percentage of potentially recruitable lung may be harmful [8]. These studies did not evaluate completely the effect of using individualised PEEP on the survival rate of patients with ARDS. The higher PEEP levels should be adjusted according to individual lung morphology. However, the strategies for setting PEEP using a plateau pressure or recruitment manoeuvre do not decrease mortality [9,10].

We designed an individual PEEP strategy using decremental PEEP titration after an alveolar recruitment manoeuvre (ARM) for each patient with ARDS. An ARM is a way to standardise the history of lung volume [11]. The objective of this study was to evaluate whether setting the PEEP using decremental PEEP titration after ARM affects the oxygenation and outcome of patients in the early stage of ARDS compared with the table-based combinations of FiO_2 _and PEEP in the ARDS network [1].

## Materials and methods

### Study population

Fifty-seven consecutive patients (35 men and 22 women) admitted to the medical intensive care unit (MICU) of Asan Medical Center, in Seoul, Korea, who were diagnosed with ARDS of various aetiologies were enrolled in the study between July 2004 and September 2006. Patient selection for the study was based on the criteria of ARDS proposed by the American–European Consensus Conference on ARDS [12]: acute onset, presence of hypoxaemia (partial arterial pressure of oxygen (PaO_2_)/FiO_2 _(PF ratio) ≤ 200 mmHg regardless of the PEEP level), bilateral and diffuse opacities seen on frontal chest x-ray and absence of left ventricular failure with pulmonary arterial occluded pressure of 18 mmHg of less.

The study protocol was approved by the institutional board of the ethics committee and written informed consent was obtained from the patients' families.

### Ventilator procedures

**Figure 1:**
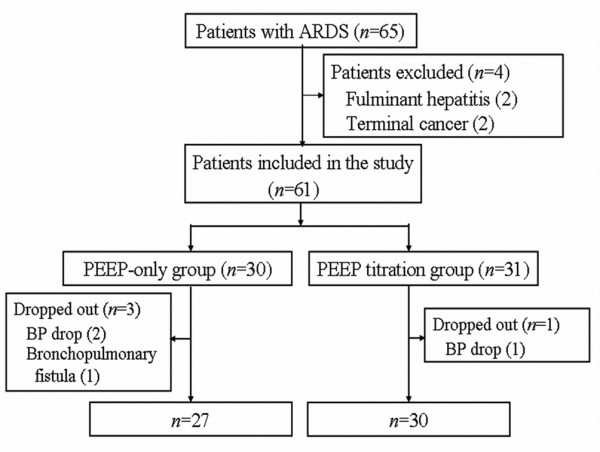
Study groups of patients. Four patients who withdrew from the study were excluded from the analysis. ARDS = acute respiratory distress syndrome; PEEP = positive end-expiratory pressure.

Patients were given ventilatory support primarily in the supine position and ventilated according to the ARDS network strategy. FiO_2_, PEEP and respiratory rate were set to achieve an arterial oxygen saturation (SaO_2_) of between 88 and 92% [2]. The target V_T _was 6 ml/kg of predicted body weight, with allowances of up to 8 ml/kg if the SaO_2 _were below 88% or less than 7.2 of arterial pH by severe hypercapnoea. The patients were randomly assigned with the use of a randomisation scheme to either the decremental PEEP titration group or the control (table-based PEEP setting) group (Figure 1).

### Patient group

#### Table-based PEEP setting (control) group

**Figure 2:**
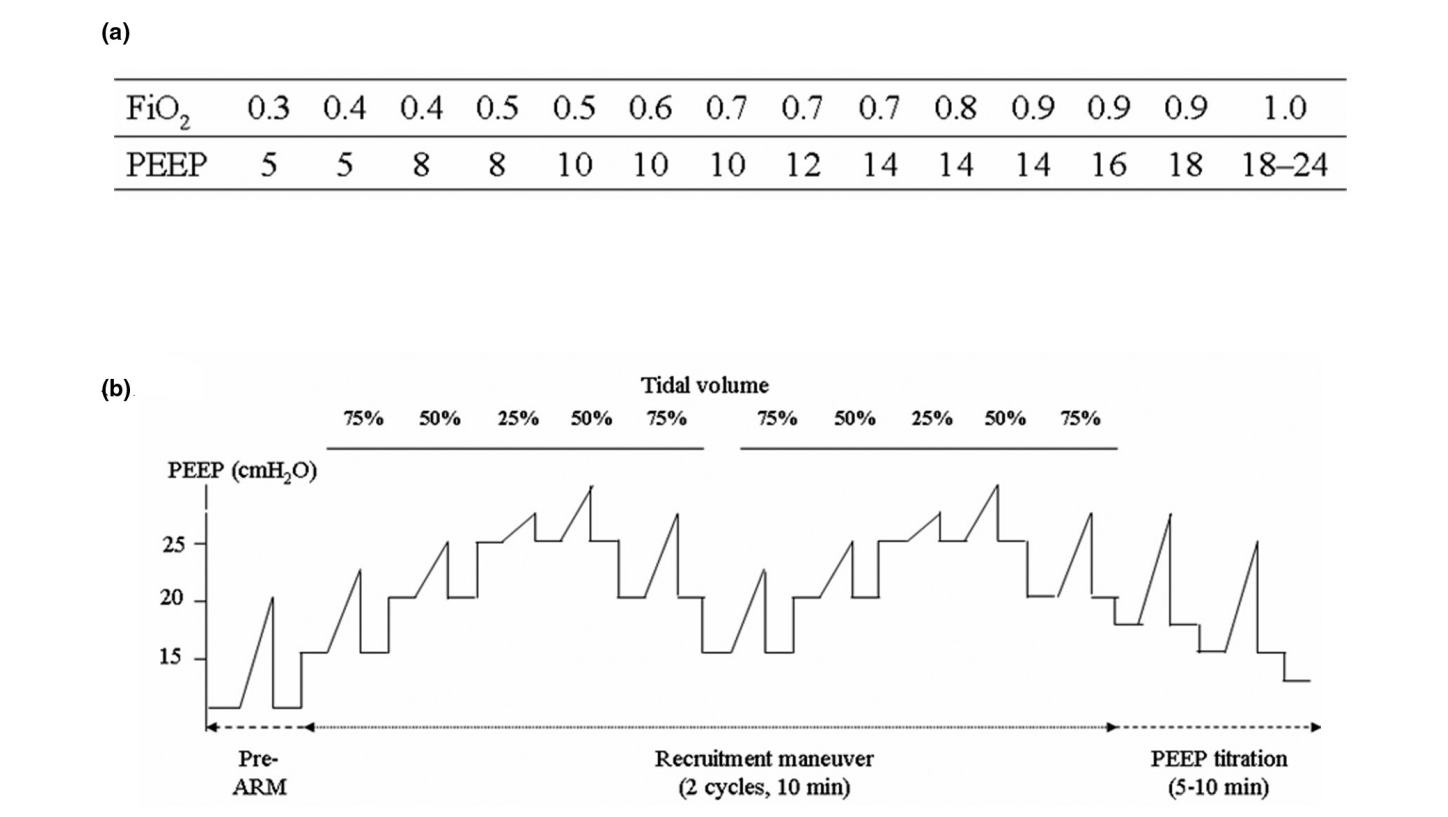
Study protocol. **(a) **Table-based positive end-expiratory pressure (PEEP) setting (control) group. **(b) **Decremental PEEP titration group after alveolar recruitment manoeuvre (ARM). FiO_2 _= inspired fracture of oxygen.

The FiO_2_-PEEP strategy has been used in previous ARDS Network studies (Figure 2). PEEP and FiO_2 _were set according to the table of lower PEEP/higher FiO_2 _combinations (the lower PEEP strategy of the ALVEOLI study), with the goal of obtaining a lower PEEP level compatible with an oxygenation target.

#### Decremental PEEP titration group

The ARM was performed immediately after enrollment in the study and was applied once a day usually in the morning for one week. The ventilatory circuit was not disconnected after ARM to avoid lung derecruitment. The ARM was also repeated when the ventilatory circuit was disconnected (incidentally or for bronchoscope) or if FiO_2 _requirement was increasing again in the patient, who showed initial improvement of oxygenation by ARM. If the weaning trial was performed within one week, the ARM was stopped earlier than the usual schedule. During the ARM, all patients were sedated and paralysed by continuous infusion of midazolam-ketamine and vecuronium bromide. No changes were made in the doses of inotropic agents or fluid infusion during the ARM.

The ARM used the extended sigh method, which is designed to gradually apply and withdraw a high distending pressure over a prolonged period. It takes about 15 to 20 minutes for two cycles of ARM according to our protocol (Figure 2) [13]. We changed the ventilatory mode from pressure-controlled mode to the volume-controlled mode during ARM. The distending pressure was determined by the delivered V_T_, PEEP increment and the pause time (0.5 seconds). During the ARM, PEEP was added from baseline to 15, 20 and 25 cmH_2_O sequentially (every 30 seconds from the baseline PEEP until 25 cmH_2_O). The V_T _was decreased by 25% from the baseline V_T _during the incremental PEEP trial phase, and then returned to baseline levels during the decremental PEEP trial phase. Therefore, the distending pressures were changed depending on the patient's V_T _and lung mechanics. However, we did not allow the peak airway pressure to go above 55 cmH_2_O during the ARM. The decremental PEEP titration at the second cycle of ARM was performed with progressive decreases in PEEP in steps of 1 cmH_2_O every 30 seconds from 20 cmH_2_O while continuously monitoring saturation and static compliance. The decrease in PEEP was continued until a decrease of more than 2% of saturation from the previous SaO_2 _and drop of static compliance was identified. This PEEP level was considered the alveolar collapsing pressure and the optimal PEEP after the ARM was set 2 cmH_2_O above this pressure. No patients showed significant arrhythmia or gross barotraumas of any type during ARM.

#### Rescue therapy

If the level of inspired oxygen was not decreased to 0.6 or oxygenation improvement was not achieved after PEEP readjustment in all patients, rescue therapies such as prone position or nitric oxide inhalation were performed.

### Outcome measures and data collection

The primary end point was improvement in oxygenation (improvement of PaO_2_/FiO_2 _(PF) ratio). The secondary end points included respiratory mechanics (PEEP and dynamic compliance), ICU stay, duration of sedatives and paralysing agents and patient outcomes (28-day mortality, 60-day mortality, duration of mechanical ventilation). Responders were defined by a 20% improvement in the PF ratio on day 1 compared with day 0 (baseline) after PEEP adjustment [14].

#### Respiratory mechanics

Airway pressure and flow were monitored continuously. V_T_, dynamic compliance (in ml/cmH_2_O), and peak airway, mean airway and minute ventilation were recorded at 30 minutes after the change of PEEP level in both groups. Because pressure control mode was the main ventilatory strategy, we monitored and compared the dynamic compliance and peak airway pressure in both group.

#### Haemodynamics and gas exchange

We collected arterial blood samples to measure partial pressure of oxygen (PO_2_), partial pressure of carbon dioxide (PCO_2_), pH, and SaO_2 _30 minutes after reapplication of PEEP. Haemodynamic variables monitored included heart rate and systolic, diastolic and mean systemic arterial pressure.

### Data analysis

Sample size calculation showed that 36 patients per group would provide 80% power at a two-sided α level of 0.05 to detect a 20% difference in the improvement of oxygenation (improvement of PF ratio).

All data were analysed using SPSS for Windows (version 11.0; SPSS Inc., Chicago, IL, USA). Statistical analyses were based on the intention-to-treat principle and involved all patients who had undergone randomisation. All values are expressed as the mean ± standard error of the mean or as the number and percentage of patients. Probability of mortality and differences between the groups were compared using a log-rank test. The chi-squared test or Fisher's exact test was used to compare categorical data, and Student's *t *test or the Mann-Whitney U test was used to compare continuous data. Significance was defined as p < 0.05.

## Results

### Characteristics of the patients

We enrolled 61 patients in the study, 30 of whom were randomly assigned to the control (table-based PEEP setting) group and 31 to the decremental PEEP titration group. Fifty-seven patients completed the study (27 in the control group and 30 in the decremental PEEP titration group) and contributed data for the analyses. Most baseline characteristics were similar in the two study groups (Table 1). The most common cause of ARDS was pneumonia. The initial ventilatory setting and severity index did not differ between the control and decremental PEEP titration groups.

**Table 1:** Baseline characteristics of the patients at enrollment

	Control group (n = 27)	Decremental PEEP titration group (n = 30)
Age (years)	62.0 ± 2.2	55.0 ± 3.7
Percentage of women	37	40
APACHE II score	20.0 ± 1.4	22.0 ± 1.1
Lung injury score	2.5 ± 0.1	2.8 ± 0.2
Tidal volume (ml/kg of predicted body weight)	8.0 ± 1.4	7.9 ± 1.9
Respiratory rate (breaths/minute)	22.0 ± 3.0	22.2 ± 3.2
Peak airway pressure (cmH_2_O)	25.9 ± 5.9	27.8 ± 5.5
Positive end-expiratory pressure (cmH_2_O)	7.0 ± 3.7	8.4 ± 3.1
Dynamic compliance (Cdyn, ml/cmH_2_O)	25.7 ± 8.1	24.3 ± 7.6
PF ratio (PaO_2_:FiO_2_)	110.8 ± 6.3	115.0 ± 8.5
Co-morbidities, n		
Haematological malignancy	3	7
Solid organ malignancy	6	4
Chronic liver disease	5	3
Connective tissue disease	2	1
Others^a^	3	3
Cause of lung injury (ARDSp:ARDSexp)	18:9	20:10
Pneumonia	15	17
Sepsis	7	7
Massive transfusion	2	1
Pulmonary alveolar haemorrhage	1	1
Others^b^	2	4

Data was presented as mean ± standard error of the mean. ^a ^including complicated diabetes mellitus (n = 4), Crohn's disease (n = 1) and AIDS (n = 1). ^b ^including leptospirosis (n = 3), near-drowning (n = 1), contusion (n = 1) and scrub typhus (n = 1). APACHE = acute physiology and chronic health evaluation; ARDSp = pulmonary ARDS; ARDSexp = extrapulmonary ARDS; FiO_2 _= inspired fracture of oxygen; PaO_2 _= partial arterial pressure of oxygen; PEEP = positive end-expiratory pressure.

### Respiratory mechanics and oxygenation

**Figure 3:**
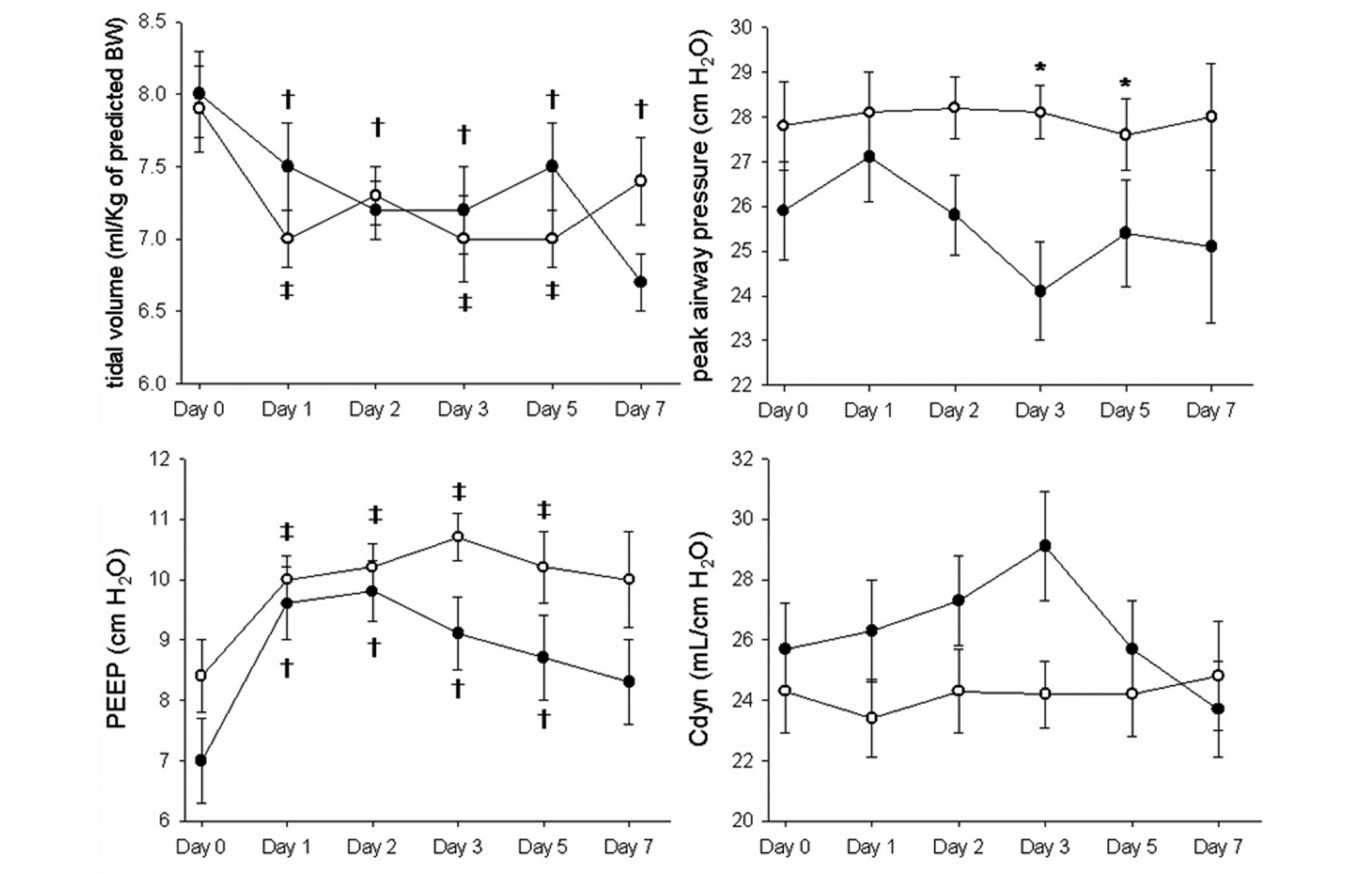
Respiratory values during the first week of treatment. The closed circles indicate the control group and the open circles denote the decremental positive end-expiratory pressure (PEEP) titration group. Values are expressed as the mean ± standard error of the mean (bars). *p < 0.05 between the control and decremental PEEP titration groups; ^†^p < 0.05 compared with day 0 in the control group; ^‡^p < 0.05 compared with day 0 in the decremental PEEP titration group.

**Figure 4:**
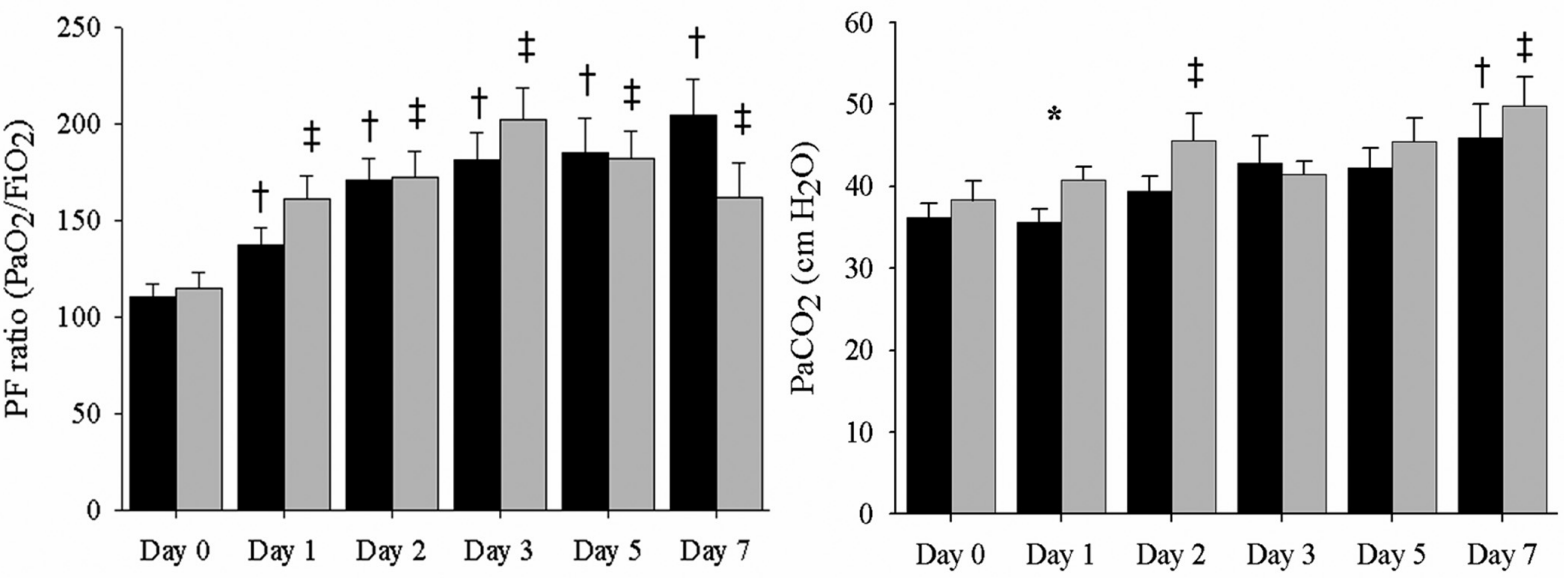
Oxygenation changes and PaCO_2 _levels during the first week of treatment. All patients showed improved oxygenation during treatment. The level of partial arterial pressure of carbon dioxide (PaCO_2_) at day 1 was increased significantly in the decremental positive end-expiratory pressure (PEEP) titration group than the control group. The black bars indicate the control group and gray bars denote the decremental PEEP titration group. Values are expressed as the mean ± standard error of the mean (bars). *p < 0.05 between the control and decremental PEEP titration groups, ^†^p < 0.05 compared with day 0 in the control group, ^‡^p < 0.05 compared with day 0 in the decremental PEEP titration group.

Figure 3 shows the ventilatory setting and respiratory variables at baseline and follow-up during the first week of treatment. The peak pressure was significantly higher on days 3 and 5 in the decremental PEEP titration group than in the control group, and the mean pressure was significantly higher in the decremental PEEP titration group because of the higher PEEP during the first week. Dynamic compliance and V_T _during the first week were similar in the two groups. Oxygenation improved compared with the baseline PF ratio in both groups (Figure 4). Initial oxygenation improved more in the decremental PEEP titration group than in the control group. The partial arterial pressure of oxygen (PaCO_2_) level was significantly higher in the decremental PEEP titration group than in the control group on day 1, suggesting increased dead space ventilation. However, the improvement of oxygenation and PaCO_2 _level were not different between the two groups during follow-up.

### Clinical outcomes

The overall mortality at 28 days was 37%. Mortality at 28 days was 33% in the control group and 40% in the decremental PEEP titration group (Table 2). Using Cox regression for 28-day mortality, the survival rate in the decremental PEEP titration group was not different (p = 0.725; hazard ratio = 1.168; 95% confidence interval = 0.493 to 2.768). However, 60-day mortality was significantly increased to 55.6% (p = 0.031) compared with 28-day mortality (33.3%) in the control group only.

**Table 2:** Clinical outcomes according to treatment group

	Control group (n = 27)	Decremental PEEP titration group (n = 30)	p value
Responder (%)	44.4	70	0.046
Clinical outcomes			
Duration of mechanical ventilation, days	15.2 ± 3.2	19.8 ± 0.5	0.380
Intensive care unit stay, days	21.4 ± 5.3	25.1 ± 5.6	0.643
Duration of paralysing agent, days	9.0 ± 2.3	11.8 ± 2.0	0.358
Duration of sedative agents, days	14.2 ± 2.4	18.7 ± 3.4	0.303
Weaning trial within seven days	8	6	
Tracheostomy	5	8	
Reintubation	5	2	
Barotrauma	3	3	
Ventilator-associated pneumonia	5	5	
Mortality, number (%)			
Mortality at 28 days	9 (33.3)	12 (40)	0.784
Death in the intensive care unit	13 (48.1)	14 (46.7)	1.000
Mortality at 60 days	15 (55.6)	14 (46.7)	0.599
Cause of in-hospital death, number (%)			
Progressive respiratory failure	8 (53.3)	8 (57.1)	
Refractory septic shock	4 (26.7)	2 (14.3)	
Hepatic failure	3 (20)	2 (14.3)	
Myocardial infarction		2 (14.3)	
Rescue therapy (%)			
Prone position	44.4	50.0	0.792
Nitric oxide inhalation	48.1	53.3	0.793

Data was presented as mean ± standard error of the mean. PEEP = positive end-expiratory pressure.

Eight patients (30%) in the control group and six patients (20%) in the decremental PEEP titration group had performed the weaning trial within a week of enrollment. The incidence of barotrauma was similar in both groups. The durations of mechanical ventilation, ICU stay and use of paralysing or sedative agents did not differ between the groups (Table 2).

## Discussion

As suggested by Lachmann more than 10 years ago [15], "open up the lung and keep the lung open" appears advantageous to recruit the lungs of patients with ARDS and to prevent subsequent lung derecruitment [12]. Clinical outcomes were similar regardless of whether lower or higher PEEP levels were used in the ALVEOLI trial [7]. The problem of the ALVEOLI trial was the higher PEEP strategy, which was table-based like the one on the current study, matched on the oxygenation target regardless of any patient-related variable. A reasonable approach to determining the appropriate level of PEEP requires maintaining PEEP-induced reopening of atelectatic areas and avoiding PEEP-induced lung overinflation. Higher than traditional PEEP levels together with lung-recruiting maneuvers seems to be a way to find an appropriate PEEP level [16].

Experimental data suggest that the effects of ARM on alveolar recruitment are transient if the preceding PEEP levels are maintained after the manoeuvre [14]. Once the alveoli have been recruited, higher PEEP levels are required to keep them aerated [17]. The right level of PEEP as an anti-derecruiting force is important in preserving the effect of the ARM. We previously reported that a sufficient level of PEEP after ARM is important as an anti-decruitment strategy [18].

We hypothesised that the decremental PEEP trial would be an appropriate method to establish the PEEP level after ARM at bedside. Because higher PEEP and the ARM performed in patients with mild lung injury may have fewer benefits and more adverse effects, we only focused on ARDS patients to address our assumption. We found that daily decremental PEEP titration after ARM showed only initial oxygenation improvement compared with the table-based PEEP method and did not improve the respiratory mechanics within a week. We performed the ARM and PEEP titration daily during the first week in the decremental PEEP titration group. However, no significant differences were observed in the 28-day mortality, ICU stay and 60-day mortality. Although the responder rate was higher in the decremental PEEP titration group than in the control group, the earlier improvement in oxygenation was not associated with increased survival rate.

Our finding showed the 60-day mortality in the control group was significantly increased compared with the 28-day mortality. We could not explain whether this finding was associated with the protective effect of ARM to the ventilator-induced lung injury. To add any relevant information, further study will be needed for the biomarkers such as proinflammatory cytokines.

The lower dynamic compliance in the decremental PEEP titration group was an unexpected result, although there was no significant difference between the two groups. We speculated that a higher peak airway pressure might affect the lower dynamic compliance in the decremental PEEP titration group than the control. A reason for this result might be related to the subjects' characteristics. Most of the patients with ARDS had pneumonia, which did not respond well to the applied PEEP [19].

We included mostly pulmonary ARDS with severe underlying diseases, which would be a common phenomenon in a university hospital MICU. That may be the reason why mortality related to progressive respiratory failure was higher in our study group. The mortality of patients with pneumonia was 43.3% and the mortality of patients without pneumonia was 29.6%. The baseline lung conditions of these patients was a higher proportion of refractory consolidation to the distending pressure which may also have influenced the PF ratio of less than 250 and PEEP levels less than 15 cmH_2_O after ARM. Talmor and colleagues reported that a low lung distending pressure was applied in the patients with a stiff chest wall [20]. We did not measure the chest-wall mechanics or the recruitable lung, so we could not address this possibility as a cause of low PF ratio.

One limitation of our study is that we could not evaluate whether recruitment manoeuvre maximised the alveolar collapse. If the recruitment effect of our ARM was not sufficient, the effect of a decremental PEEP trial might be insufficient. Borges and colleagues reported that a peak airway pressure of more than 60 cmH_2_O could recruit the collapsed lung [21]. Another limitation is that our indicators of oxygen saturation and static compliance to find an appropriate PEEP level might be insensitive to detect the collapsing pressure of the lung. Finally, small sample size in a single centre limits the power of the study outcomes.

## Conclusion

The daily decremental PEEP titration after ARM did not show the persistent improvement of oxygenation, the respiratory mechanics and the mortality rate of patients with ARDS compared with the table-based PEEP setting. Further investigations are needed to find the correct level of PEEP in ARDS with reference to chest wall compliance and alveoli mechanics.

## Key messages

• The daily decremental PEEP titration after ARM method did not show a persistent improvement of oxygenation.

• Respiratory mechanics such as dynamic compliance, V_T _and PEEP were not significantly different between the daily decremental PEEP titration after ARM group and the table-based PEEP setting group.

• The daily decremental PEEP titration after ARM without the reference of lung mechanics did not reduce the dependence of ventilator or mortality rate of patients with ARDS compared with the table-based PEEP setting.

## Abbreviations

ALI: acute lung injury; ARDS: acute respiratory distress syndrome; ARM: alveolar recruitment manoeuvre; FiO_2_: inspired fracture of oxygen; MICU: medical intensive care unit; PEEP: positive end-expiratory pressure; PaCO_2_: partial arterial pressure of carbon dioxide; PaO_2_: partial arterial pressure of oxygen; PCO_2_: partial pressure of carbon dioxide; PO_2_: partial pressure of oxygen; SaO_2_: arterial oxygen saturation; V_T_: tidal volume.

## Competing interests

The authors declare that they have no competing interests.

## Authors' contributions

JWH recruited patients, analysed the data and wrote the manuscript. HJ and HSC helped to recruit patients and analysed the data. SBH and CML recruited patients and interpreted the data. YK designed the study, interpreted the data and wrote the paper. All authors read and approved the final manuscript.
